# The Gut–Prostate axis in chronic Prostatitis/Chronic Pelvic pain syndrome: Mechanisms, evidence gaps, and biomarker-stratified translation

**DOI:** 10.1016/j.isci.2026.116935

**Published:** 2026-07-29

**Authors:** Shuo Pang, Yuxin Liu, Kaixin Shi, BaiLin Yi, Shiming Wang, Yingwei Bi, Jianbo Wang

**Affiliations:** 1 Department of Urology, The First Affiliated Hospital of Dalian Medical University, Dalian 116011, China; 2 Department of Urology, The Second Affiliated Hospital of Dalian Medical University, Dalian 116011, China

**Keywords:** microbiota-gut-brain axis, Th17 cells, immunometabolism, systemic inflammation, prostatitis

## Abstract

Chronic prostatitis/chronic pelvic pain syndrome (CP/CPPS) is a heterogeneous disorder traditionally framed around local prostate, pelvic-floor, neurological, and psychosocial factors. Emerging evidence suggests that gut dysbiosis may contribute to CP/CPPS in a subset of patients through barrier dysfunction, immune priming, microbial metabolites, and neuroimmune modulation. We propose a Dual-Hit Permissive Model in which gut-derived systemic immunometabolic priming must converge with prostate-local or pelvic permissive cues before organ-localized inflammation and chronic pain can be sustained. We distinguish direct human CP/CPPS evidence from animal prostatitis models, contextual prostate-disease evidence, and hypothesis-generating mechanisms. Ectopic mucosal addressins, prostate-specific vascular “address codes,” and microbial-prostate molecular mimicry remain testable hypotheses rather than established mechanisms. Future work should prioritize biomarker-stratified, target-engagement studies to validate immunometabolic, tissue-localization, and neuroimmune checkpoints before gut-directed interventions are broadly translated into CP/CPPS care.

## Graphical abstract

**Figure:**
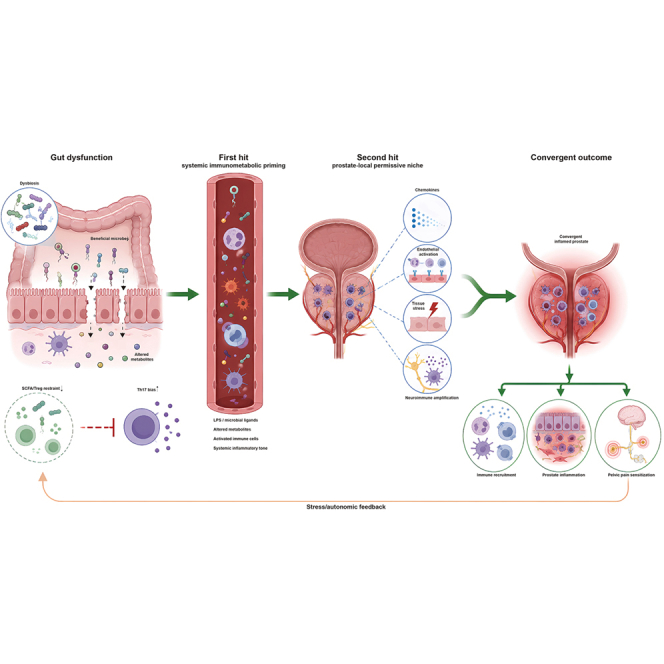

## Teaser

Gastroenterology; Urology; Systems biology

## Introduction

Chronic prostatitis/chronic pelvic pain syndrome (CP/CPPS) is a common and disabling urological disease in men, characterized by pelvic pain, urinary symptoms, and sexual dysfunction, substantially impairing patients’ quality of life.^1^^,^^2^ The etiology and pathogenesis of CP/CPPS are complex and remain incompletely understood, involving immune, neurological, and psychosocial factors. Growing evidence indicates that interactions between the gut microbiota and the prostate are closely related to CP/CPPS pathogenesis.^3^^,^^4^

**Figure 1:**
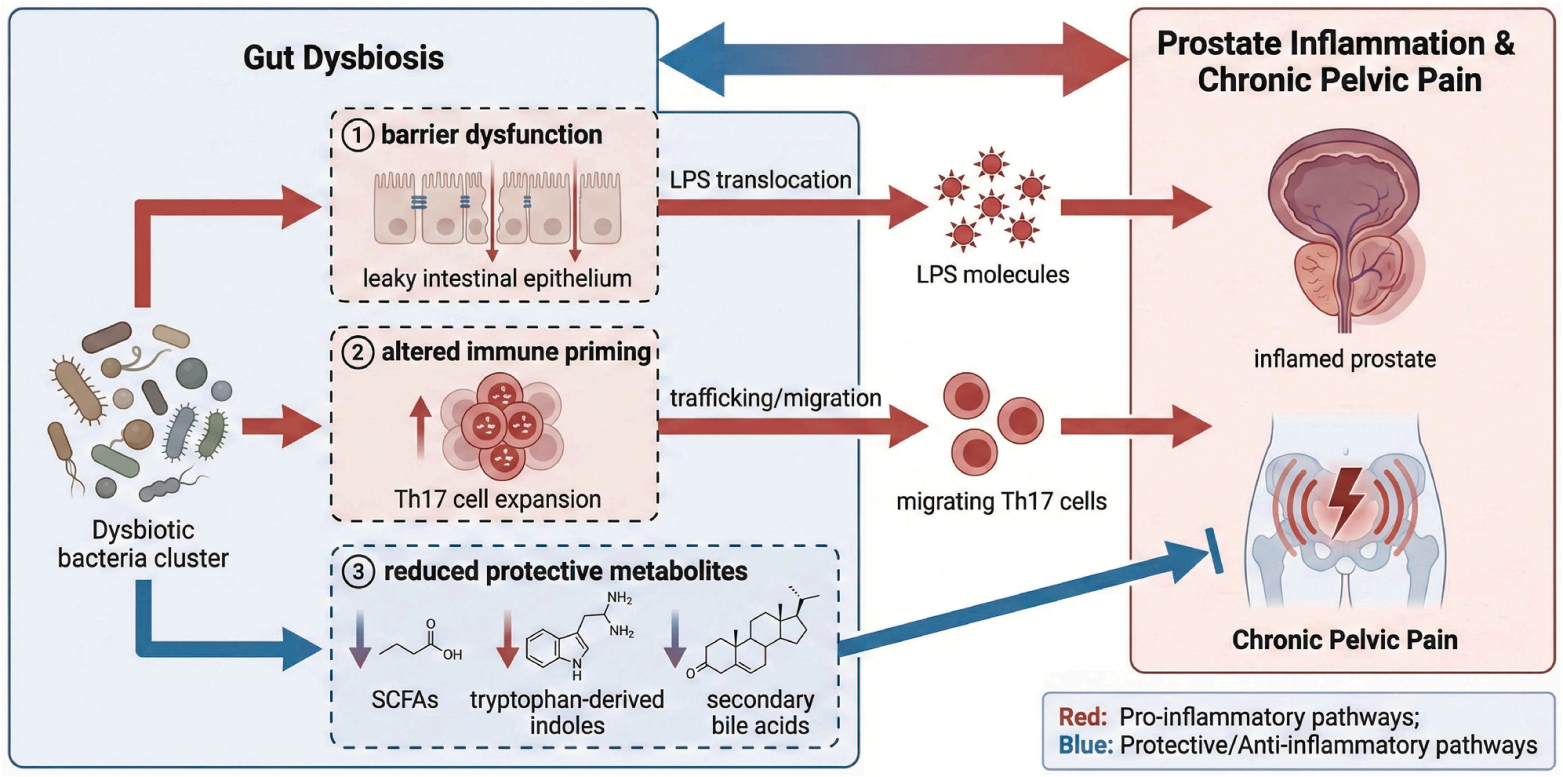
Overview of the gut-prostate axis in CP/CPPS Gut dysbiosis is linked to three major, evidence-weighted pathways: (1) intestinal barrier dysfunction and LPS translocation, (2) altered immune priming with Th17 expansion and potential immune-cell trafficking, and (3) reduced protective microbial metabolites, including SCFAs, tryptophan-derived indoles, and secondary bile acids. These pathways may converge on prostatic inflammation and chronic pelvic pain, with bidirectional interactions between gut dysbiosis, prostate-local inflammation, and pain/stress-associated neuroimmune outputs. In this overview, gut microbiota alterations are supported by human CP/CPPS observational or intervention-associated studies and animal prostatitis models; barrier dysfunction, LPS translocation, and Th17 expansion are supported mainly by animal prostatitis evidence with limited human biomarker support; metabolite-related nodes are supported by animal, contextual, and limited human observational evidence; and gut-to-prostate immune trafficking remains hypothesis-generating in human CP/CPPS because direct lineage-tracing or shared-TCR evidence is lacking. Red indicates pro-inflammatory pathways and blue indicates protective or anti-inflammatory pathways.

**Figure 2:**
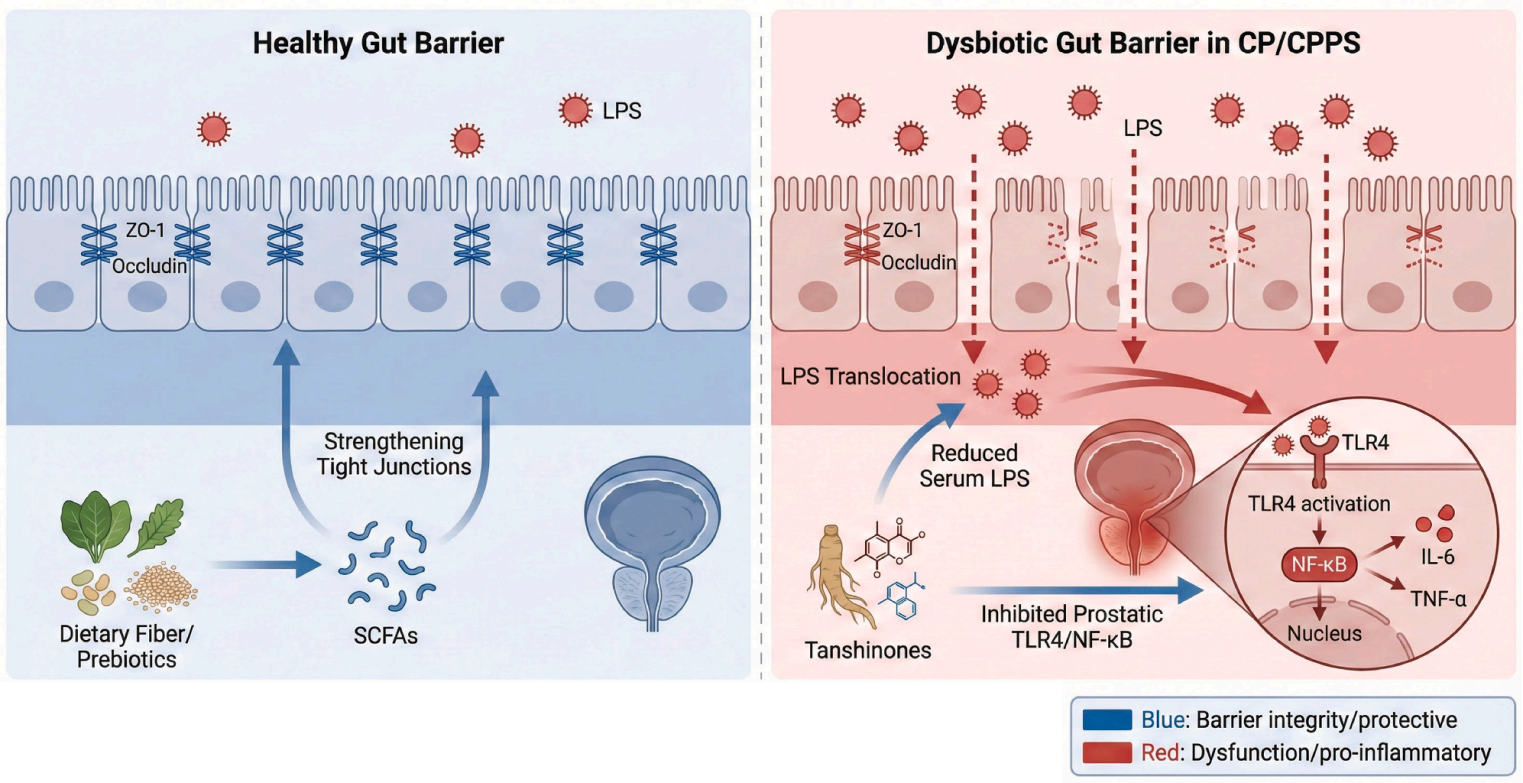
Mechanisms of intestinal barrier dysfunction and endotoxemia in the gut-prostate axis of CP/CPPS Under homeostatic conditions, epithelial tight junction proteins such as ZO-1 and occludin help maintain gut barrier integrity, and SCFAs generated from dietary fiber or prebiotics support barrier function and limit excessive immune activation. In dysbiosis-associated CP/CPPS models, disruption of tight junctions may increase intestinal permeability, allowing LPS and related microbial ligands to translocate into the circulation. These circulating signals may amplify prostate-local innate inflammatory programs through TLR4-NF-κB activation, promoting inflammatory mediators such as IL-6 and TNF-α. Tanshinone-related interventions are shown as examples of preclinical strategies reported to reduce serum LPS and inhibit prostatic TLR4/NF-κB signaling in experimental settings. Evidence labels for this figure should be interpreted as follows: barrier dysfunction, LPS translocation, and TLR4-NF-κB activation are supported mainly by animal prostatitis evidence, with limited human biomarker support; SCFA-mediated barrier protection is supported by animal and contextual mucosal-immunology evidence; and the LPS-TLR4-NF-κB axis is presented as a plausible inflammatory amplifier rather than a proven causal mechanism in human CP/CPPS. Blue denotes barrier-protective or anti-inflammatory pathways; red denotes barrier dysfunction or pro-inflammatory signaling.

**Figure 3:**
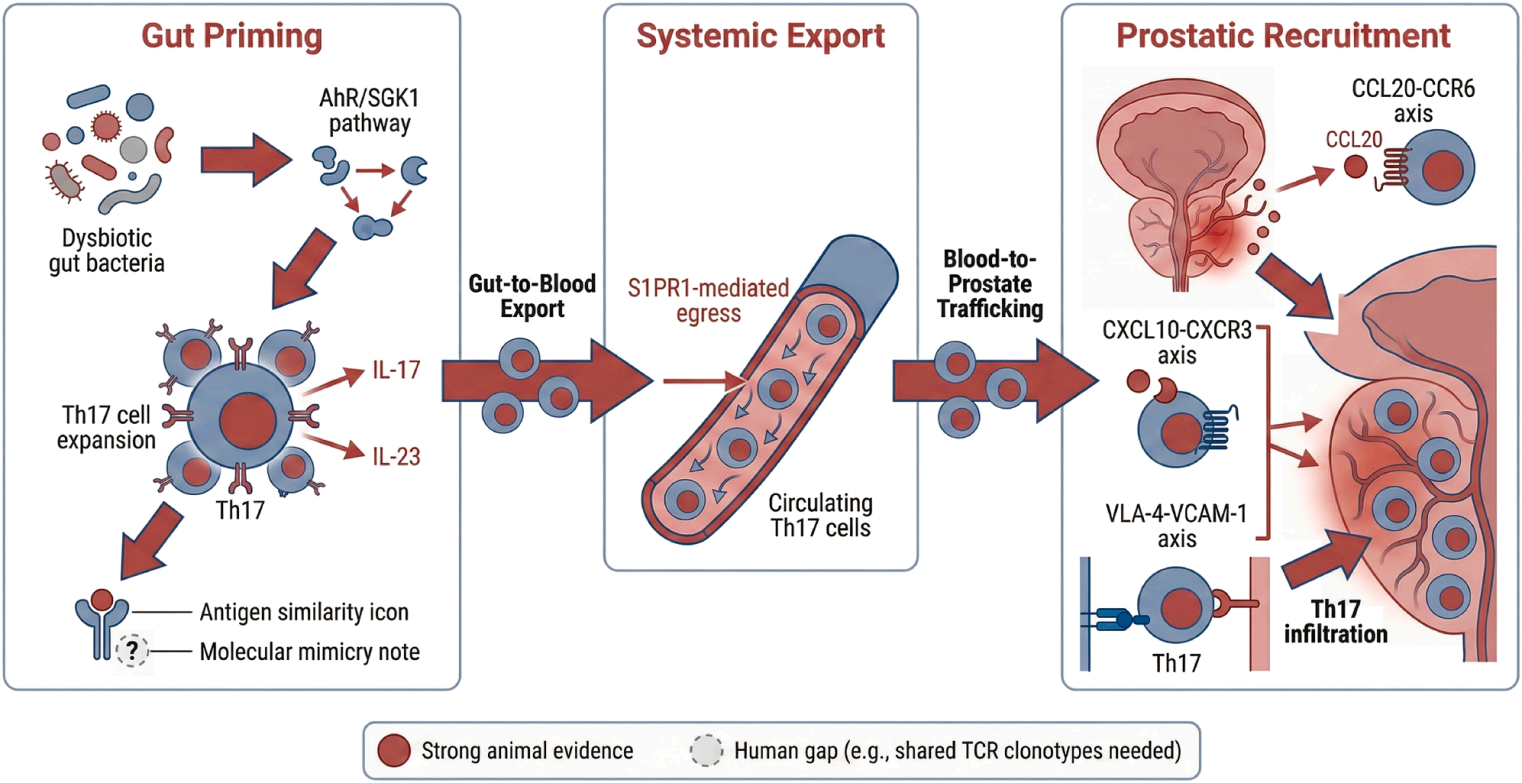
Gut-to-prostate immune priming and trafficking pipeline in CP/CPPS This figure summarizes a plausible but incompletely validated framework linking gut dysbiosis to prostate-local immune recruitment. In the gut-priming phase, dysbiosis may skew mucosal immune programs toward Th17 expansion, including the high-salt–5-HIAA–AhR–SGK1/FOXO1 axis, which is supported mainly by animal prostatitis evidence. Putative antigen similarity and molecular mimicry are shown as indirect and hypothesis-generating mechanisms because specific microbial-prostate antigen pairs remain poorly defined. In the systemic-export phase, S1PR1-mediated egress is shown as a model-supported mechanism by which mucosa-primed effector cells may enter the circulation. In the prostatic-recruitment phase, chemokine and adhesion pathways, including CCL20-CCR6, CXCL10-CXCR3, and VLA-4-VCAM-1, identify recruitment-compatible checkpoints in prostatitis models. VCAM-1/VLA-4 is presented as a general inflammatory docking mechanism rather than as a prostate-specific homing code. These pathways do not establish that prostate-infiltrating immune cells in human CP/CPPS are gut-derived. The evidence key in this figure should be interpreted as follows: solid elements denote dominant support from animal prostatitis models, whereas dashed elements indicate major human evidence gaps. Direct human validation would require paired gut–blood–prostate immune profiling, shared TCR clonotypes, mucosal-homing signatures, or lineage-tracing evidence. Representative evidence, evidence levels, and interpretation boundaries are summarized in Table 1.

**Figure 4:**
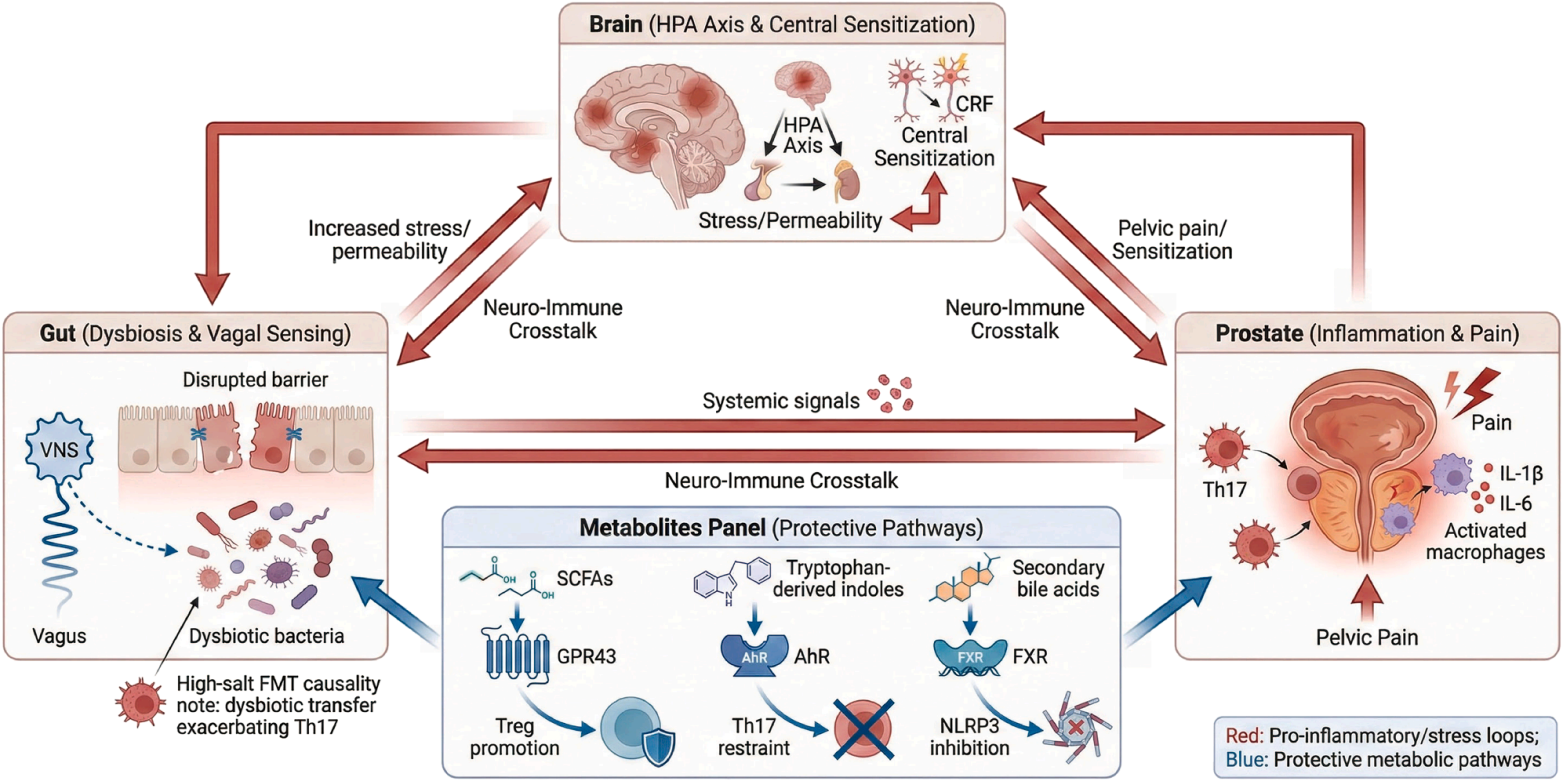
Metabolic cross-talk and neuroimmune modulation in the gut–prostate axis of CP/CPPS This schematic integrates dysbiosis-associated barrier disruption, systemic inflammatory signaling, microbial metabolite pathways, and pain-related neuroimmune feedback within the gut–immune–prostate–pain network. The gut panel includes disrupted barrier function, vagal sensing, and high-salt FMT-related animal evidence indicating that dysbiotic transfer can enhance Th17-associated inflammatory responses in prostatitis models, rather than direct proof of the same causal pathway in human CP/CPPS. The metabolite panel summarizes protective or regulatory pathways involving SCFAs-GPR43-Treg promotion, tryptophan-derived indoles-AhR-Th17 restraint, and secondary bile acids-FXR-NLRP3 inhibition. The brain/HPA-axis, autonomic, and central-sensitization modules are shown as modulatory loops that may influence gut permeability, neuroimmune crosstalk, and pelvic pain amplification. In the prostate panel, Th17-associated inflammation, activated macrophages, IL-1β/IL-6 signaling, and pelvic pain are presented as downstream inflammatory and pain-related outputs. Red denotes pro-inflammatory or stress-related loops; blue denotes protective metabolic pathways. Representative evidence, evidence levels, and interpretation boundaries are summarized in Table 1. Across Figures 1, 2, 3, and 4, evidence labels indicate the dominant evidence type supporting each depicted node, including direct human CP/CPPS evidence, animal prostatitis evidence, contextual prostate-disease evidence, and hypothesis-generating mechanisms. Representative evidence, evidence levels, and interpretation boundaries are summarized in Table 1.

Relevant studies suggest that gut disorders can affect remote organs, including the prostate, through systemic inflammation, immune cell migration, and circulating microbial metabolites.^3^^,^^5^^,^^6^^,^^7^ This paradigm is supported by emerging clinical observations and mechanistic research. Compared with healthy controls, the intestinal microbiota diversity of patients with CP/CPPS is lower. Interventions targeting the gut microbiome can alleviate prostatitis and pain in animal models and have shown similar trends in some population studies.^8^^,^^9^ Other diseases of the urinary system, including lower urinary tract symptoms and prostate cancer (PCa), have been confirmed to be related to disorders of the intestinal microbiome.^10^^,^^11^ A unifying hypothesis is that an imbalance in intestinal homeostasis may trigger or exacerbate prostatic disease. In CP/CPPS, abnormal gut microbiota may contribute to systemic immune and metabolic perturbations that could lower the threshold for pelvic inflammation in susceptible patients.^4^^,^^12^ This review examines how intestinal barrier dysfunction, intestinal immune education, chemokine-driven cell homing, molecular mimicry, and microbial metabolites jointly influence prostatitis. We integrate findings from human studies, animal prostatitis models, and contextual prostate-disease literature to evaluate which components of the gut–prostate axis are supported by direct evidence and which remain hypothesis-generating. We also discuss how causal inference, methodological variability, and patient heterogeneity should shape future microbiome-targeted research. To improve conceptual clarity, Figure 1 presents an evidence-weighted overview of the proposed gut–prostate axis in CP/CPPS. The schematic summarizes a stepwise progression from gut dysfunction to systemic immunometabolic priming, prostate-local permissiveness, and ultimately prostatic inflammation and chronic pelvic pain. It also highlights potential feedback from pain- and stress-associated autonomic outputs to gut permeability and microbial ecology. Figures 2, 3, and 4 then expand the barrier, immune-trafficking, and metabolite/neuroimmune modules in greater mechanistic detail.

These observations raise a central localization problem. Gut dysbiosis, intestinal barrier dysfunction, and systemic immune activation are not unique to CP/CPPS, yet CP/CPPS manifests with prostate- and pelvis-centered inflammation and pain. A gut–prostate framework therefore needs to explain not only how intestinal perturbations generate systemic immunometabolic priming, but also why these signals become clinically amplified in the prostate and pelvic pain network in only a subset of patients. This argues against a simple “gut dysbiosis causes prostatitis” model and motivates a framework that integrates systemic priming with organ-local permissive conditions.

To address this gap, we propose the Dual-Hit Permissive Model as a testable framework rather than as an established causal pathway. The first hit refers to gut-derived systemic immunometabolic priming, including intestinal barrier dysfunction, microbial ligand translocation, T helper 17 (Th17)/regulatory T cells (Treg) imbalance, and altered microbial metabolite signaling. The second hit refers to prostate-local or pelvic permissive cues, including chemokine gradients, endothelial activation, tissue stress, blood-prostate barrier perturbation, and neuroimmune amplification. These second-hit factors differ in evidentiary strength: chemokine and adhesion programs are supported mainly by animal prostatitis models and inflammatory readouts, whereas microtrauma, hypoxia, hormonal imbalance, and the proposed gut–prostate vascular “address code” remain hypothesis-generating mechanisms requiring direct validation in human CP/CPPS. Thus, the model is intended to organize existing evidence, define evidence boundaries, and generate falsifiable predictions for future studies.

Accordingly, this Review focuses on non-malignant inflammatory CP/CPPS and on gut–prostate mechanisms centered on intestinal barrier dysfunction, immune-cell migration, immunometabolic signaling, and neuroimmune modulation. Evidence from benign prostatic hyperplasia and PCa is discussed only when it clarifies conserved biological pathways, and is treated as contextual background rather than direct clinical evidence for CP/CPPS. The literature-search strategy and evidence-appraisal approach are described in the STAR Methods.

## The dual-hit permissive model as a falsifiable framework

With the localization problem and evidence hierarchy outlined above in mind, we define the Dual-Hit Permissive Model as a testable framework rather than as an established causal pathway. The model does not assume that gut dysbiosis alone causes CP/CPPS, nor does it imply that all patients have a gut-driven disease subtype. Instead, it proposes that prostate-centered inflammation and pain may arise in a subset of patients when systemic gut-derived immunometabolic priming converges with a prostate-local or pelvic permissive niche.

### First hit: Systemic priming

The first hit refers to gut-derived systemic immunometabolic priming, including intestinal barrier dysfunction, endotoxemia-associated signaling, Th17/Treg imbalance, altered microbial metabolite profiles, and broader systemic inflammatory tone. These processes are biologically plausible and partly supported by animal prostatitis models and limited human biomarker observations, but they are not prostate-specific. Therefore, the first hit should be viewed as a systemic permissive state rather than as a sufficient explanation for prostate-local disease.

### Second hit: Local permissiveness

The second hit refers to prostate-local or pelvic permissive conditions that may determine whether systemic immune stress becomes amplified within the prostate and pelvic pain network. These conditions may include prostate-local chemokine gradients, inflammatory endothelial activation, vascular docking programs, tissue-stress signals, blood-prostate barrier perturbation, and neuroimmune amplification. Importantly, these components differ in evidentiary strength: chemokine and adhesion programs are supported mainly by animal prostatitis models and inflammatory readouts, whereas microtrauma, hypoxia, hormonal imbalance, MAdCAM-1, mucosa-like addressins, and the proposed gut–prostate vascular “address code” remain hypothesis-generating mechanisms requiring direct validation in human CP/CPPS.

### Temporal flexibility and thresholds

The model does not require a single fixed temporal order. Gut-derived systemic priming may precede and sensitize the host to prostate-local inflammatory amplification, whereas prostate-local tissue stress, pelvic-floor dysfunction, infection-related inflammation, or neurogenic pain may also precede gut changes and subsequently amplify dysbiosis through stress, diet, medication exposure, or autonomic outputs. Thus, the model is better interpreted as a threshold-based permissive framework than as a linear sequence. Persistent CP/CPPS-like pathology may be more likely when systemic priming and prostate-local permissive signals both exceed a threshold required for immune recruitment, retention, inflammatory amplification, or pain-network sensitization.

### Falsifiable predictions

This framework generates several experimentally testable predictions. Patients with a gut-driven CP/CPPS phenotype should show systemic immunometabolic priming, such as increased permeability or endotoxemia-associated markers, altered microbial metabolite profiles, or Th17/Treg imbalance. Prostate-local samples should show recruitment-compatible inflammatory signals, such as chemokine gradients, endothelial activation markers, or neuroimmune inflammatory readouts. If gut-educated immune cells directly contribute to prostate inflammation, paired gut-blood-prostate profiling should reveal shared TCR clonotypes, mucosal-homing signatures, or related transcriptional states in prostate-infiltrating immune cells.

Conversely, if prostate-infiltrating immune cells lack mucosal-homing features, if prostate-local inflammation occurs independently of systemic priming, or if gut-directed interventions improve symptoms without altering target biomarkers, the model would require revision. In this way, the Dual-Hit Permissive Model is intended not as a fixed disease explanation, but as a falsifiable framework for organizing evidence and guiding future biomarker-stratified studies.

## Existing CP/CPPS paradigms and the place of the gut–prostate axis

CP/CPPS should be understood as a heterogeneous syndrome rather than as a single gut-driven inflammatory disease. Established clinical phenotyping frameworks, such as UPOINT, already recognize urinary, psychosocial, organ-specific, infection-related, neurological/systemic, and pelvic-floor tenderness domains.^13^ This multidomain view helps explain why patients with similar prostate-local findings can differ substantially in pain distribution, symptom burden, comorbid symptoms, and treatment response.^14^

Neurogenic inflammation and central sensitization provide complementary explanations for persistent pelvic pain. Peripheral nociceptive input from the prostate, pelvic floor, or adjacent pelvic organs may sensitize dorsal root ganglia, spinal dorsal horn circuits, and central pain-processing networks. Brain functional and anatomical changes in CP/CPPS further support involvement of central pain-processing circuits.^15^ These mechanisms are not microbiome-specific, but they may interact with gut-derived immune or metabolic signals by lowering pain thresholds and amplifying neuroimmune responses.

Psychosocial contributors, including stress, anxiety, depression, catastrophizing, sleep disturbance, and reduced physical activity, can also influence symptom severity, pain persistence, and treatment response.^16^ These factors are also relevant to microbiome interpretation because they may alter diet, medication exposure, autonomic output, intestinal permeability, and microbial ecology, thereby acting as confounders or mediators rather than simple downstream consequences.

Accordingly, the gut–prostate axis should be viewed as a complementary framework rather than as a replacement for neurogenic, central-sensitization, psychosocial, pelvic-floor, autoimmune, or local-inflammatory models of CP/CPPS. It may be most relevant to a subset of patients in whom gut-derived immunometabolic priming converges with prostate-local inflammatory permissiveness and pain-network amplification.^17^ This positioning clarifies why microbiome-directed interventions should be tested in biomarker-defined subgroups rather than generalized to all patients with CP/CPPS. Thus, the proposed Dual-Hit Permissive Model should be viewed as an extension of, rather than a replacement for, traditional CP/CPPS paradigms. Compared with infection-based or purely local inflammatory models, this framework emphasizes how systemic gut-derived immunometabolic priming may converge with prostate-local permissive cues and neuroimmune amplification to explain disease persistence and patient heterogeneity. Its main novelty is to provide a testable, biomarker-stratified structure for linking systemic immune-metabolic signals to organ-local symptom expression.

## Intestinal barrier dysfunction and microbial translocation

A healthy intestinal barrier prevents excessive translocation of bacterial products, such as lipopolysaccharide (LPS), into the systemic circulation. In dysbiosis-associated states, increased intestinal permeability may allow microbial products to disseminate systemically and increase inflammatory exposure in peripheral tissues, including the prostate.^18^^,^^19^ Mechanistically, this provides a plausible route by which gut-derived microbial ligands could amplify innate immune activation through pathways such as LPS-TLR4-NF-κB signaling.

Animal prostatitis models provide the strongest mechanistic support for this barrier-first concept. In the rodent CP/CPPS model, total tanshinone, a herbal anti-inflammatory compound, significantly reduced serum LPS levels and improved related indicators such as barrier tight-junction integrity, while alleviating pain and reducing prostatitis.^20^^,^^21^ After decontamination treatment, the original protective effect was no longer observed, whereas fecal microbiota transplantation (FMT) partially restored the protective phenotype, suggesting that disease remission depended at least in part on microbiota-mediated improvement of barrier function.^22^ In the autoimmune prostatitis model, antibiotic mixtures altered the gut microbiota and bile acid profile, which was consistent with enhanced intestinal barrier function and reduced LPS leakage, thereby alleviating prostatitis.^23^^,^^24^

The main translational gap is whether this complete barrier-LPS-prostate inflammatory sequence operates causally in human CP/CPPS. Restoring barrier integrity can inhibit the LPS-TLR4 signaling pathway in the prostate, and tanshinone-related studies have shown parallel reductions in circulating LPS and prostatic TLR4/NF-κB activation.^20^^,^^25^ Elevated circulating LPS levels and increased intestinal permeability have also been reported in refractory prostatic diseases and correlate with disease severity.^18^^,^^26^ In addition, fermentable fiber may increase short-chain fatty acid levels and strengthen epithelial tight junctions, and this has been associated with prostatitis alleviation in preclinical models.^11^^,^^27^ Thus, intestinal barrier integrity is best viewed as a plausible upstream amplifier of gut–prostate inflammation, but its causal role in human CP/CPPS requires longitudinal and target-engagement validation.

Findings from PCa studies further support the possibility that barrier dysfunction and endotoxemia influence prostate-related inflammation. However, the immunosuppressive tumor microenvironment and treatment-induced immune remodeling differ from those in CP/CPPS. Therefore, these PCa-derived observations should be interpreted as mechanistic background rather than direct evidence for CP/CPPS. In PCa, androgen deprivation and chemotherapy are associated with increased intestinal permeability and endotoxemia, linking barrier dysfunction to disease-related systemic inflammation.^28^ Conversely, an intestine enriched in probiotics enhances barrier function. Supplementation with inosine derived from Akkermansia improved mucosal integrity in mice and reduced serum LPS levels. It has been reported that inosine can inhibit prostate tumor growth via the LPS/NF-κB/AR (androgen receptor) axis.^26^^,^^29^These observations in PCa indicate a relatively conserved mechanistic sequence—“barrier → microbial ligand/metabolite → innate immune activation,” but whether this sequence constitutes the causal mechanism in CP/CPPS remains to be verified.

Importantly, the LPS-TLR4-NF-κB sequence should be interpreted according to evidence strength. In CP/CPPS, direct human evidence for barrier dysfunction and endotoxemia remains limited and largely biomarker-based, whereas stronger mechanistic support comes from animal prostatitis models and contextual prostate-disease studies. Therefore, this pathway is presented as a plausible inflammatory amplifier rather than as a proven causal mechanism in human CP/CPPS. Throughout this Review, we distinguish direct CP/CPPS evidence from animal-model evidence and contextual evidence from other prostate diseases, as summarized in Table 1.

**Table 1:** Evidence hierarchy, major findings, and interpretation boundaries for key human, animal, and contextual evidence supporting the gut-prostate axis in CP/CPPS

Mechanistic node	Representative evidence/major findings	Dominant evidence label	Evidence level	Limitations/interpretation boundary
Intestinal barrier dysfunction/microbial translocation (LPS-TLR4-NF-kB)	barrier/permeability and endotoxemia observations, tanshinone/FMT and antibiotic-mixture prostatitis models, and contextual PCa/BPH data support a barrier-microbial ligand-innate inflammation sequence.^18^^,^^19^^,^^20^^,^^21^^,^^22^^,^^23^^,^^24^^,^^25^^,^^26^^,^^27^^,^^28^^,^^29^	human CP/CPPS (limited biomarker) + animal prostatitis + contextual prostate disease	level 2–3; level 5 for PCa/BPH context	plausible inflammatory amplifier; the complete barrier-LPS-prostate causal sequence is not established in human CP/CPPS. Figure 2 nodes should be interpreted as human/animal/contextual evidence rather than proven causal mechanisms.
Gut immune education/Th17-Treg imbalance and high-salt-5-HIAA-AhR-SGK1/FOXO1 axis	animal and contextual evidence links gut dysbiosis, high-salt exposure, altered 5-HIAA/AhR signaling, SGK1/FOXO1 activation, and Th17 skewing; CP/CPPS immune imbalance is discussed as limited human/contextual support.^1^^,^^30^^,^^31^^,^^32^^,^^33^^,^^34^^,^^35^	animal prostatitis/high-salt model; limited human immune observations	level 2–3	supports Th17-skewing plausibility in animal prostatitis models; does not prove intestinal origin of prostate-infiltrating T cells or confirm this axis as a human CP/CPPS mechanism. Figure 3/4 nodes should be labeled as Animal prostatitis or limited human evidence.
Bile-acid-FXR-NLRP3-IL-17 immune regulation	antibiotic-induced microbiota disturbance and bile-acid remodeling are linked to FXR-NLRP3-IL-17 regulation and reduced Th17-driven prostatitis; Eriocalyxin B-associated bile-acid remodeling also supports metabolite-sensitive macrophage regulation.^6^^,^^36^^,^^37^^,^^38^^,^^39^	animal prostatitis	level 3	mechanistic support for metabolite-sensitive immune regulation in EAP; relevance to human CP/CPPS remains inferential until paired stool, serum, and prostate-local metabolomic/immune readouts are available.
SCFAs-GPR43/HDAC-Treg programs and epithelial barrier support	SCFAs are discussed as regulatory metabolites supporting Treg programs, anti-inflammatory signaling, and barrier integrity; reduced SCFA-producing taxa and metabolic comorbidity are linked to CP/CPPS or contextual prostate-disease observations.^11^^,^^27^^,^^40^^,^^41^^,^^42^^,^^43^^,^^44^^,^^45^	human CP/CPPS (observational) + animal/contextual mucosal immunology	level 2–4	biologically plausible anti-inflammatory and barrier-supporting pathway; patient-level therapeutic response prediction remains unvalidated. Figure 4 should present this as animal/contextual or limited human biomarker evidence.
IL-17A-epithelial pyroptosis-dsHMGB1-M1 macrophage amplification	Ma et al. showed that IL-17A-induced prostate epithelial pyroptosis releases dsHMGB1 and promotes M1 macrophage polarization through Pfkp/Jak2-Stat1-dependent metabolic reprogramming in EAP.^46^	animal prostatitis	level 3	concrete EAP support for a Th17-epithelial injury-macrophage amplification loop; does not establish gut-derived initiation or human CP/CPPS causality.
Chemokine-driven recruitment: CCL20-CCR6, CXCL10-CXCR3, CXCL12-CXCR4/AMD3100	EAP and bacterial prostatitis studies support CCL20-CCR6, CXCL10-CXCR3, and CXCR4/AMD3100-sensitive recruitment or expansion programs; human prostatic inflammatory readouts support recruitment-compatible signaling.^47^^,^^48^^,^^49^^,^^50^^,^^51^	animal prostatitis + human CP/CPPS inflammatory readout	level 2–3	supports a prostate recruitment-compatible inflammatory program; dominant trafficking hierarchy and responsive patient subgroup in human CP/CPPS remain undefined. Figure 3 nodes should be interpreted separately by evidence type.
S1P/S1PR1-dependent lymphocyte egress	FTY720/S1PR1 modulation suppressed experimental autoimmune prostatitis, supporting S1PR1-dependent lymphocyte egress as a systemic effector-cell availability checkpoint.^52^	animal prostatitis	level 3	plausible checkpoint for systemic effector-cell availability; not prostate-specific and not validated as a human CP/CPPS therapeutic target.
Vascular docking: VCAM-1/VLA-4	VLA-4/VCAM-1 is discussed as a conserved inflammatory adhesion mechanism that may facilitate extravasation and retention when combined with prostate-local chemokine signals and tissue stress.^47^^,^^48^^,^^49^^,^^50^^,^^51^^,^^52^	animal/general inflammatory evidence	level 3–4	general inflammatory docking mechanism that can facilitate tissue entry but cannot by itself explain prostate selectivity because VCAM-1 is broadly inducible in inflamed tissues.
MAdCAM-1/ectopic mucosal addressins/gut-prostate vascular address code	no direct human CP/CPPS prostate-endothelial evidence is currently available in the manuscript; this concept is retained only as a second-hit, experimentally testable hypothesis.	hypothesis-generating	level 5	unverified but testable hypothesis. Should be depicted, if at all, with dashed/hypothesis labeling; requires endothelial profiling, spatial transcriptomics, or immunostaining in well-phenotyped CP/CPPS samples.
Molecular mimicry/microbial-prostate antigenic cross-reactivity	EAP/autoimmune-like observations and microbiota-modulation studies support plausibility, but specific microbial-prostate antigen pairs remain poorly defined.^4^^,^^35^^,^^53^^,^^54^^,^^55^^,^^56^	indirect/hypothesis-generating	level 4–5	biologically plausible but incomplete. Human antigen pairs and T cell specificity remain unvalidated; should be framed as indirect, incomplete, and hypothesis-generating rather than an established mechanism.
Causal inference and microbiome-prostatic disease genetics	two-sample Mendelian randomization studies link gut microbial features with prostatic disease outcomes, including prostatitis/BPH/PCa-related outcomes, sex hormones, metabolites, and proteins.^57^^,^^58^	human genetic causal-inference evidence	level 2; not intervention evidence	MR strengthens causal plausibility and reduces some confounding/reverse causation, but cannot identify actionable strains, metabolites, prostate-local mechanisms, immune-cell origin, or treatment response.
Human microbiome signatures, treatment-associated shifts, and biomarker-guided stratification	human CP/CPPS studies report fecal microbial signatures with non-invasive biomarker potential and intervention-associated microbiome shifts accompanying symptom changes in Li-ESWT/pre-post cohorts; these remain unvalidated for predicting probiotic, dietary, or microbiome-targeted response.^44^^,^^45^^,^^59^^,^^60^^,^^61^^,^^62^	human CP/CPPS interventional/longitudinal + observational evidence	level 1–2 for intervention-associated microbiome shifts and observational biomarkers; not validated response biomarker	level 1-type evidence is limited to treatment-associated or longitudinal microbiome observations and does not establish gut-directed causal therapy. Biomarkers should be framed as investigational; fecal microbial signatures may support future stratification, but fecal calprotectin or microbiome profiles should not be presented as validated CP/CPPS treatment-response markers.

Evidence labels used in Figures 1, 2, 3, and 4 and this table: human CP/CPPS, direct human observational/interventional evidence in CP/CPPS; animal prostatitis, EAP, bacterial prostatitis, FMT, antibiotic, high-salt, or related prostatitis model evidence; Contextual prostate disease, BPH/PCa evidence used only for conserved-pathway plausibility; hypothesis-generating, unverified mechanisms requiring direct validation.

Evidence levels: level 1, direct human CP/CPPS interventional or longitudinal evidence (including treatment-associated microbiome changes, but not necessarily causal proof of gut-directed therapy); level 2, human CP/CPPS observational/biomarker or genetic causal-inference evidence; level 3, animal prostatitis model evidence; level 4, general mechanistic immunology evidence; Level 5, contextual BPH/PCa evidence or hypothesis-generating evidence.

Abbreviations: AhR, aryl hydrocarbon receptor; BPH, benign prostatic hyperplasia; CP/CPPS, chronic prostatitis/chronic pelvic pain syndrome; EAP, experimental autoimmune prostatitis; FMT, fecal microbiota transplantation; LPS, lipopolysaccharide; MR, Mendelian randomization; PCa, prostate cancer; SCFAs, short-chain fatty acids.

To mechanistically consolidate this barrier-first model, Figure 2 summarizes how dysbiosis-associated tight-junction disruption enables LPS translocation and endotoxemia, and how downstream prostatic TLR4-NF-κB signaling can amplify inflammatory outputs relevant to CP/CPPS. The schematic also highlights candidate barrier-supporting levers (e.g., SCFA-linked epithelial reinforcement) and an endotoxemia-to-prostate signaling axis that can be interrogated with target-engagement biomarkers.

## Immune education and T cell priming in the gut

The gastrointestinal tract is a major site for immune cell education. Intestinal antigen-presenting cells continuously sample microbial antigens and activate naive T cells in gut-associated lymphoid tissues, enabling these cells to acquire effector phenotypes and homing programs. Dysregulated T cell responses are closely associated with CP/CPPS, particularly expansion of proinflammatory Th17 cells relative to Treg.^1^^,^^30^ This provides the mechanistic basis for considering the gut as a potential site of systemic immune priming.

Animal models provide the strongest support for microbiota-dependent Th17 skewing. Some commensal bacteria can induce Th17 differentiation in the intestinal mucosa, and in prostatitis models, intestinal dysbiosis disrupts T cell balance. A high-salt diet has been shown to alter the microbiota, reduce beneficial bacteria, and increase bacteria that degrade serotonin, thereby promoting Th17 expansion and aggravating prostatitis.^31^^,^^32^ Mechanistically, high salt intake alters microbial production of tryptophan metabolites, including 5-hydroxyindoleacetic acid (5-HIAA), which can act on AhR in CD4^+^ T cells and promote Th17 differentiation through the SGK1-FOXO1 pathway^33^^,^^34^ This led to Th17 accumulation in the prostate and exacerbated inflammation and pain in mice.^32^ This 5-HIAA-AhR-SGK1/FOXO1 pathway should therefore be interpreted as animal prostatitis evidence for high-salt-induced Th17 skewing rather than as a confirmed mechanism in human CP/CPPS.

Additional metabolite-sensitive pathways may counterbalance or amplify Th17 programs. Antibiotic-induced microbiota disturbance can increase colonic bile acid levels and activate FXR, thereby inhibiting the proinflammatory NLRP3-IL-17 axis and limiting Th17 generation.^36^^,^^37^ This was associated with reduced Th17 infiltration in the prostate and alleviation of autoimmune prostatitis.^37^^,^^38^ Recent experimental autoimmune prostatitis (EAPs) work further strengthens the link between Th17-centered inflammation and myeloid amplification in prostatitis, while also illustrating that this evidence remains model-based. Ma et al. showed that IL-17A-induced pyroptosis of prostate epithelial cells can promote the release of double-stranded HMGB1, which enhances M1 macrophage polarization through Pfkp/Jak2-Stat1-dependent metabolic reprogramming.^46^ This provides concrete support for a Th17-epithelial injury-macrophage amplification loop in EAP, but it does not establish that the initiating Th17 program originates from the gut in human CP/CPPS.

Regulatory programs may also be relevant. Treg expansion is generally considered protective, and SCFAs such as butyrate can enhance Treg differentiation and immunomodulatory function. Clinical observations suggest that patients with CP/CPPS with metabolic syndrome often exhibit more severe symptoms, possibly related to reduced SCFA-producing capacity and fewer Treg cells.^27^^,^^40^ Experimental prostatitis studies also suggest that, when chemotactic signals are present, activated lymphocytes from intestinal-associated lymphoid tissue can migrate to inflamed prostate tissue.^47^^,^^63^

The key evidence gap is source attribution in humans. Shared Th17/Th1-biased inflammatory patterns, gastrointestinal comorbidity, and prostate-local chemokine signals are consistent with a gut-immune contribution, but they do not establish the intestinal origin of prostate-infiltrating immune cells. Direct evidence would require paired gut–blood–prostate profiling, shared TCR clonotypes, mucosal-homing receptor signatures, or lineage-tracing approaches capable of linking gut-educated immune populations to prostate-local inflammation. Until such evidence is available, the gut-origin trafficking model should be interpreted as a plausible but unproven mechanism in human CP/CPPS.

## Chemokine-driven immune trafficking from gut to prostate

The migration of immune cells between mucosal sites is governed by gradients of tissue chemokines and by adhesion programs. These two signal types act together to determine cellular tissue selectivity. In intestinal-associated lymphoid tissues, activation enables lymphocytes to express canonical mucosal homing receptors such as CCR9 and integrin α4β7. After these cells enter the bloodstream, encountering strong peripheral recruitment signals can alter their homing trajectories and redirect them to sites of extraintestinal inflammation.

In experimental prostatitis, the inflamed prostate can express chemokines capable of capturing circulating effector cells, including CCL20 under inflammatory conditions.^47^ In the autoimmune prostatitis model, prostate CCL20 expression synchronized with Th17 cell accumulation, consistent with recruitment via the CCL20-CCR6 axis. Animal experiments showed CCR6^+^/CCR7^+^ memory T cell subsets in the inflamed prostate, and their characteristics matched the migration pattern of mucosa-associated lymphoid tissue.^48^ In addition to lymphocytes, migration of innate immune cells may contribute. In an Escherichia coli-induced prostatitis model, intestinal-derived macrophages migrated to the prostate and exacerbated the local inflammatory response by releasing IL-1β.^30^^,^^49^

In patients, an inflammatory chemokine environment in prostatic secretions supports active recruitment. Elevated IFN-γ and CXCL10 in prostatic fluid are consistent with a Th1-biased microenvironment and with the potential recruitment of CXCR3^+^ T cells.^50^ These signals alone do not prove that the recruited cells originate from the intestine or carry intestinally induced imprints. Rather, they indicate that when a systemic pool of effector T cells is present, the local prostate provides conditions that promote T cell recruitment and infiltration.

Additional EAP evidence supports the relevance of chemokine-directed T cell recruitment in prostatitis. Hu et al. reported that pharmacological blockade of CXCR4 with AMD3100 alleviated prostatic inflammation and pelvic pain in EAP mice, accompanied by suppression of Th17 cell differentiation and oxidative stress.^51^ Together with CCL20-CCR6 and CXCL10-CXCR3 observations, these findings support the concept that chemokine pathways can regulate pathogenic immune-cell recruitment or expansion in prostatitis models. However, they should be interpreted as animal-model evidence rather than direct proof that the same trafficking hierarchy dominates in human CP/CPPS.

Lymphocyte trafficking depends not only on chemokines but also on lymphocyte availability and vascular docking. S1PR1-dependent lymphocyte egress is crucial for maintaining the size of the circulating lymphocyte pool. Previous studies have shown that blocking S1PR1 alleviated Escherichia coli-induced inflammatory prostatitis. This finding suggests there may be regulatory interactions within lymphoid tissue that limit the egress of pathogenic immune cells.^52^ At the tissue-entry step, integrins such as α4β1 (VLA-4) can bind VCAM-1 on inflamed prostate endothelium, facilitating extravasation and retention.

Thus, VCAM-1/VLA-4 should be viewed as a general inflammatory docking mechanism rather than as a prostate-specific homing code. Its relevance to CP/CPPS depends on whether it operates together with prostate-local chemokine gradients, tissue stress signals, or other still-unverified endothelial programs. Because VCAM-1 can be upregulated in many inflamed tissues, this mechanism alone cannot confer a specific homing preference for the prostate over other potential target organs. We therefore treat the proposed gut–prostate vascular “address code” as an unverified but experimentally testable hypothesis. Under chronic pelvic stress, local hypoxia, urinary reflux, or other tissue-stress conditions, prostate endothelial cells might theoretically acquire adhesion or glycosylation features that favor the docking of mucosa-educated immune cells. However, direct evidence for MAdCAM-1, mucosa-like addressins, or related ectopic anchoring platforms in human CP/CPPS prostate tissue is currently lacking. Future studies should test this hypothesis using endothelial profiling, spatial transcriptomics, and immunostaining for candidate addressins and adhesion molecules in well-phenotyped CP/CPPS samples.

Taken together, the available evidence supports a plausible but incompletely proven cascade in which mucosal immune priming may expand Th17/Th1-biased effector pools, while prostate-local chemokine gradients and adhesion programs may promote immune-cell recruitment and retention. However, the source attribution of prostate-infiltrating immune cells remains a critical unresolved gap. It remains necessary to determine whether these cells carry definitive molecular signatures of mucosal education or instead reflect local expansion, systemic inflammation, or other non-gut-derived immune processes. Addressing this gap will require paired single-cell transcriptomics and TCR sequencing across intestinal, blood, and prostate-local samples.

This hierarchical evidence framework provides a rationale for the plausibility of these therapeutic directions. CCR6/CCL20 antagonists, S1P modulators, or blockade of the downstream IL-17/IL-1β pathway are expected to reduce inflammatory cell recruitment and limit their expansion. These approaches should remain candidate strategies based on mechanistic rationale. Given pathway redundancy and potential risks to systemic immunity, careful validation through rigorous experiments and clinical evidence is required.

To consolidate the gut-to-prostate immune priming/trafficking concept into a single, testable framework, Figure 3 summarizes the sequential checkpoints from gut Th17 skewing (e.g., AhR/SGK1-linked programs) to systemic export (S1PR1-mediated egress) and prostate recruitment (CCL20-CCR6, CXCL10-CXCR3, and VLA-4–VCAM-1), highlighting where strong animal evidence exists and where direct human proof remains a key gap (e.g., shared TCR clonotypes).

## Molecular mimicry between microbial and prostate antigens

One intriguing mechanism for gut-induced prostatic inflammation is molecular mimicry, wherein immune responses against gut microbial antigens cross-react with homologous self-antigens in the prostate. The immunological features of CP/CPPS are consistent with an autoimmune process. In many cases, evidence of active infection is lacking, yet immune-cell infiltration persists, and autoantibodies targeting prostate tissue can be detected.^4^^,^^53^ In CP/CPPS, one hypothesis proposes that structurally similar molecules contribute to pathogenesis. Certain microbial peptides produced by the intestinal or urinary microbiota resemble prostate-specific antigens, acid phosphatases, or related proteins in sequence or structure, thereby inducing T cell sensitization in genetically susceptible individuals and triggering subsequent immune responses. These T cells then return to the prostate and attack host tissue. Even after the initial microbial triggers are cleared, inflammation persists for an extended period. Evidence indicates that prostate antigen immunity can induce EAP. Further observations revealed that, when co-reared with mice carrying specific intestinal microorganisms, the onset and progression of EAP accelerated, which may reflect the adjuvant effect of microbes or the contribution of cross-reactive antigens to autoimmune amplification.^4^^,^^35^ Recent work by Yu et al. demonstrated that altering the gut flora with antibiotics alleviated EAP and decreased Th17 cells, as discussed, implying the inciting antigenic stimulus may have been microbial.^35^^,^^54^ Although molecular mimicry is biologically plausible in CP/CPPS, the specific microbial-prostate antigen pairs remain poorly defined. Reports of potential cross-reactivity between microbial antigens and prostate-related peptides should therefore be interpreted as indirect and hypothesis-generating evidence rather than as established proof of a gut-derived autoimmune mechanism. Future studies should combine microbial metagenomics, antigen discovery, peptide-MHC mapping, and TCR specificity assays to determine whether cross-reactive microbial antigens contribute to prostate-local immune activation. Intestinal dysbiosis may also lower the threshold for autoimmune-like inflammation by altering Treg-mediated tolerance and mucosal immune balance. In EAP models, interventions such as FMT or probiotics have been associated with increased Treg responses and reduced prostate tissue injury.^55^^,^^56^ These findings support the broader plausibility that microbiota-dependent immune regulation can influence prostate-directed inflammation, but they do not demonstrate that molecular mimicry is the dominant or initiating mechanism in human CP/CPPS. Clinical observations that symptoms may fluctuate after gastrointestinal infections or antibiotic exposure should likewise be interpreted cautiously, because such associations may reflect altered microbial ecology, immune tone, medication effects, or stress-related changes rather than antigen-specific cross-reactivity. Overall, molecular mimicry should be presented as a biologically plausible but indirect and incompletely validated mechanism that requires antigen-discovery, peptide-MHC mapping, and TCR-specificity studies before it can be considered established in CP/CPPS.^4^^,^^54^

## Microbial metabolites as immune modulators (SCFAs, tryptophan, bile acids)

Microbial metabolites that enter the bloodstream are central mediators of communication between the intestine and distant organs. These molecules include short-chain fatty acids, tryptophan-derived indole compounds, and secondary bile acids generated through microbial fermentation and biotransformation.^11^^,^^64^ In CP/CPPS, metabolite-mediated immune regulation may shape systemic immune tone by influencing T cell differentiation, myeloid activation, and mucosal barrier function.^11^^,^^41^

SCFAs represent the best-characterized anti-inflammatory metabolite class. Butyrate and propionate can promote Treg programs and anti-inflammatory signaling through G protein-coupled receptors such as GPR43 and through epigenetic mechanisms such as HDAC inhibition.^42^^,^^43^ Previous studies reported that the butyric acid-producing potential of the intestinal microbiota in patients with CP/CPPS decreased. In patients with CP/CPPS and concurrent fertility problems, the abundance of SCFA-producing taxa, such as Faecalibacterium and Eubacterium, was reduced and associated with elevated proinflammatory cytokines. Conversely, improving butyrate availability through dietary regulation or microbiota remodeling has been associated with reductions in prostatitis and oxidative stress indicators as well as symptom improvement.^44^^,^^45^

Tryptophan-derived metabolites provide additional immunomodulatory input through AhR-related pathways. Because the high-salt-5-HIAA-AhR-SGK1/FOXO1-Th17 axis has been discussed above as animal prostatitis evidence, this section focuses on the broader principle that microbial metabolites can reshape immune balance rather than repeating the Th17 mechanism in detail.^32^^,^^33^

Bile acid signaling also influences T cell balance, inflammasome activity, and myeloid polarization. In EAP, alterations in the secondary bile acid pool and FXR signaling were associated with Th17/Treg imbalance.^36^^,^^37^ Intervening in this pathway inhibited NLRP3 inflammasome activity and IL-17 production and improved prostatitis.^37^^,^^38^ These bile-acid-FXR-NLRP3 findings provide mechanistic support for metabolite-sensitive immune regulation in EAP, but their relevance to human CP/CPPS remains inferential until paired stool, serum, and prostate-local metabolomic and immune readouts are obtained. Bile acids may also influence macrophage polarization. In the EAP mouse model, Eriocalyxin B treatment altered the intestinal bile acid profile and was accompanied by a shift in prostate macrophages from pro-inflammatory M1 to anti-inflammatory M2.^57^^,^^65^

Overall, microbial metabolites may influence the balance between persistent and resolving inflammation by modulating Th17-driven inflammatory amplification, regulatory immune networks, epithelial barrier function, and myeloid polarization. However, these effects should be interpreted pathway-by-pathway because the evidence base differs substantially between human observational studies, animal prostatitis models, and contextual prostate-disease evidence.^11^^,^^41^

Interactions between metabolites and the immune system have also been observed in other prostate diseases. Although these studies provide mechanistic clues, their conclusions are difficult to extrapolate directly to CP/CPPS because of the disease-specific immune environment. For example, intestinal microbiota can regulate vitamin and polyamine metabolites (such as folic acid and biotin), thereby influencing immune regulation and altering the functional state of prostate cells.^66^ Gut bacteria can contribute to systemic androgen-related pools, including production of androgen analogs through enzymes such as desmolase.^67^^,^^68^ In advanced PCa, specific gut and urinary microbes (e.g., Clostridium scindens, Propionimicrobium) have been implicated in generating androgens that may sustain tumor growth despite castration therapy.^69^ These cancer-focused studies do not provide direct evidence for CP/CPPS, but they converge on a common principle: Microbial metabolites can access the prostate and influence local processes, manifesting as modulation of immune responses and alterations in the endocrine or metabolic environment.^11^^,^^36^^,^^37^^,^^38^^,^^40^^,^^41^^,^^42^^,^^43^

## Microbiota shifts in CP/CPPS and prostate inflammation

Gut microbiome dysbiosis has been reported in CP/CPPS, with early human studies suggesting reduced microbial diversity and a community structure that differs from healthy controls, although cohort sizes remain modest and clinical phenotypes are heterogeneous.^59^^,^^60^ These changes align with typical gut microbiota patterns observed in chronic inflammatory conditions, which are characterized by reduced diversity, loss of symbiotic bacteria with protective roles, and expansion of opportunistic pathogens. However, studies have reported inconsistent microbiota signatures for CP/CPPS. Therefore, a standardized sampling protocol, strict control of confounders such as diet, antibiotic use, and irritable bowel syndrome, and longitudinal study designs are necessary to improve the reliability of causal inference.^9^^,^^10^^,^^59^^,^^60^ Animal models provide stronger evidence for mechanistic studies. Multiple studies show that experimentally induced prostatitis is typically accompanied by alterations in the composition and function of the intestinal microbiota, and interventions that reduce prostatitis severity often produce a partial recovery of the intestinal microbiota structure and metabolite profile.^55^^,^^70^ A berberine-based intervention study reported that CPPS induction was associated with increased Escherichia-Shigella and Oscillibacter and decreased Prevotella and Bifidobacterium, and that microbiota transfer experiments supported a microbiome-dependent component of symptom modulation.^70^ At the translational medicine level, human intervention studies suggest that the microbiome contributes to differences in therapeutic response. In clinical trials, low-intensity shock wave therapy was associated with symptom improvement, and measurable changes in gut microbiome diversity and enrichment of anti-inflammatory metabolic pathways were observed, while nonresponders typically present with an unfavorable baseline microbiome configuration.^44^^,^^45^ Genetic causal-inference approaches, particularly Mendelian randomization (MR), provide a complementary way to evaluate whether gut microbial features may contribute to prostate-related outcomes while reducing some forms of confounding and reverse causation. For example, Liu et al. used a two-sample MR design to investigate associations between gut microbiota and common prostatic diseases and reported genetically supported links between specific microbial taxa and prostate-disease outcomes.^57^ However, MR evidence should be interpreted cautiously. It can support causal plausibility at the population-genetic level, but it does not identify the actionable microbial strains, metabolites, immune-cell trajectories, or prostate-local mechanisms responsible for disease expression. Nor can it establish that prostate-infiltrating immune cells originate from the gut. Therefore, MR findings should be viewed as hypothesis-strengthening evidence that complements, but does not replace, longitudinal microbiome profiling, intervention studies, and paired gut–blood–prostate immune analyses. Finally, the urine microbiome has also attracted attention in CP/CPPS, but its functional significance remains unclear. Because urine microorganisms often overlap with intestinal symbionts, study designs must be sufficiently rigorous to distinguish true urinary-tract ecological changes from artifacts caused by downstream contamination or sampling.^71^^,^^72^

In PCa, multiple studies report disease- and treatment-associated shifts in gut and/or urinary microbial composition and function, with proposed links to systemic inflammation, endocrine metabolism, and therapeutic responsiveness.^73^^,^^74^^,^^75^^,^^76^ In BPH, microbiome alterations have been associated with metabolic-inflammation phenotypes that may influence prostatic growth and symptom severity, again suggesting that prostatic disease states can be embedded within broader host-microbiome networks.^77^^,^^78^ However, the immune landscapes of CP/CPPS, BPH, and PCa differ: CP/CPPS involves nonmalignant inflammation primarily characterized by pain; BPH is associated with metabolic changes related to growth and aging; and PCa features tumor-driven immune remodeling. Therefore, any extrapolation of microbe-related characteristics or mechanisms must be based on disease-specific evidence.^32^^,^^75^^,^^76^^,^^77^^,^^78^ In conclusion, the emerging CP/CPPS literature indicates that gut microbiota dysbiosis is a potential regulatory feature of disease biology. However, the field currently requires coordinated multi-omics approaches, mechano-anchored models, and stratified clinical trials to distinguish microbial groups or functions that are pathogenic from those that are merely associated, and to identify which patient subgroups are most likely to benefit from microbiome-targeted interventions.^59^^,^^60^

## Causality, reverse causation, and methodological variability in microbiome studies

A central limitation of current CP/CPPS microbiome research is that most human evidence remains associative. Human studies have reported altered microbial diversity and community structure in CP/CPPS, but cohort sizes remain modest, clinical phenotypes are heterogeneous, and reported microbial signatures are not always consistent.^59^^,^^60^ Cross-sectional differences in microbial diversity, taxonomic composition, or predicted metabolic function cannot determine whether dysbiosis contributes to CP/CPPS, results from CP/CPPS, or reflects shared upstream confounders.^9^^,^^10^^,^^59^^,^^60^ Reverse causation is particularly relevant in CP/CPPS because chronic pelvic pain, psychological stress, sleep disturbance, reduced physical activity, altered diet, and repeated medication exposure may themselves reshape the gut microbiome and intestinal barrier function.^79^^,^^80^ Therefore, gut dysbiosis should not be interpreted as an upstream driver in all patients, but rather as a potentially contributory, secondary, or bidirectionally interacting factor depending on the clinical context.

Important confounders also limit causal interpretation. Diet, salt intake, fiber intake, body mass index, metabolic syndrome, irritable bowel syndrome, gastrointestinal inflammation, prior or repeated antibiotic exposure, proton pump inhibitor use, probiotics, geographic background, and lifestyle variables can all influence gut microbial profiles.^9^^,^^10^^,^^59^^,^^60^ Methodological variability further complicates comparison across studies, including differences in stool versus urine or expressed prostatic secretion sampling, sequencing platforms, taxonomic resolution, batch effects, contamination control, and bioinformatic pipelines. Urine microbiome findings require particular caution because urinary microorganisms may overlap with intestinal symbionts, and study designs must distinguish true urinary-tract ecological changes from contamination or sampling artifacts.^71^^,^^72^ These issues may partly explain why human CP/CPPS microbiome signatures remain heterogeneous and sometimes contradictory.

Future studies should therefore move beyond simple case-control taxonomic comparisons. Stronger causal inference will require longitudinal cohorts with standardized sampling, careful control of diet and medication exposure, paired stool-blood-urine or prostate-local readouts, metabolomics, immune phenotyping, and prespecified clinical endpoints.^9^^,^^10^^,^^59^^,^^60^ MR can strengthen causal plausibility while reducing some forms of confounding and reverse causation, but it cannot identify actionable microbial strains, metabolites, immune-cell trajectories, or prostate-local mechanisms.^57^ Microbiota-depletion and recolonization models, FMT-based transfer experiments, and biomarker-stratified intervention trials can further test causality and target engagement, but their interpretation should remain model- or subgroup-specific rather than generalized to all patients with CP/CPPS.^22^^,^^34^^,^^35^^,^^39^ Only convergent evidence across longitudinal human studies, mechanistic animal experiments, genetic causal-inference analyses, and target-engagement trials can determine whether a microbiome signal is pathogenic, compensatory, or merely associated with CP/CPPS.

## Insights from animal models vs. human studies

A central interpretive issue in this field is that mechanistic strength and disease specificity do not always align. Rodent EAP and bacterial prostatitis models are valuable for testing immune pathways, temporal sequence, and intervention effects, but they do not fully reproduce the clinical heterogeneity, chronic pain experience, microbiome diversity, or psychosocial context of human CP/CPPS. Conversely, human CP/CPPS studies provide disease relevance but are often cross-sectional, small, and vulnerable to confounding by diet, antibiotics, metabolic status, and comorbid gastrointestinal conditions. Therefore, throughout this Review, animal evidence is used to support mechanistic plausibility, whereas direct human CP/CPPS evidence is required before a pathway is described as disease-defining.

There is a growing synergy between animal models and human studies in elucidating the gut–prostate axis, yet important differences exist. Animal models (rodent prostatitis models, primarily) have been invaluable for mechanistic dissection. They allow controlled perturbation of the microbiome and immune system that is not feasible in humans. Germ-free or antibiotic-treated mice serve as key tools for causal inference. Experimental studies have shown that when antibiotics altered the composition of the intestinal microbiota, the early acute inflammation in EAP decreased, further supporting that the intestinal microbiota can regulate the onset and progression of prostatitis.^35^^,^^54^ Gnotobiotic mice colonized with specific bacteria have demonstrated that certain strains can either exacerbate or protect against prostatitis.^81^ Diet-induced models, including high-salt, high-fat, protein-restriction, or caloric-restriction paradigms, further illustrate that metabolic context can modify prostatitis severity through microbiome-dependent immune or metabolite pathways, as discussed in the immune-priming and metabolite sections.^33^^,^^35^ Animal studies also enable tissue analysis at early time points, revealing, for instance, that gut permeability increases precede prostate immune cell infiltration—a temporal sequence hard to capture in patients. Moreover, experiments in mice have underscored a brain–gut–prostate connection, where stress-induced changes in gut flora impacted neuroinflammation and pelvic pain.^33^ These findings prompt investigation of neuromodulators (such as vagal tone or substance P) that link gut and pelvic nerves, a frontier that animal models are poised to explore. To integrate these neuro-immune loops with immunometabolic control points, Figure 4 summarizes how stress/HPA-axis activation and autonomic outputs can feedback on gut permeability and dysbiosis, while microbiota-derived metabolites (SCFAs, tryptophan-derived indoles, and secondary bile acids) engage host receptors (e.g., GPR43, AhR, and FXR) to modulate Th17/Treg balance and inflammasome-linked inflammation, thereby shaping prostatic inflammatory amplification and pain sensitization in CP/CPPS. On the other hand, human studies provide essential validation and highlight aspects that murine models might not replicate. For instance, humans have much more diverse microbiomes and longer lifespans, which could influence chronicity of prostatitis. The first clinical microbiome studies in patients with CP/CPPS, though small, have confirmed dysbiosis and suggested correlations between specific bacteria and symptoms.^6^^,^^9^ Findings from animal models do not always translate to clinical practice. For example, Lactobacillus often improves symptoms in mouse prostatitis models, but probiotic trials in patients with CP/CPPS have produced highly variable results. This discrepancy may reflect the high heterogeneity of the human microbiome and environmental factors such as diet. In addition, some mouse strains with autoimmune genetic susceptibility spontaneously develop prostatitis, a phenomenon attributed to immune cross-reactivity linked to the intestinal microbiota. By contrast, human CP/CPPS generally lacks a clear autoimmune etiology, implying that its onset may depend on specific triggers or genetic background. Thus, animal models can only approximate the relevant processes within a limited range. Taken together, the two lines of evidence converge on a key conclusion: Interactions between the microbiome and the immune system are likely an important underlying mechanism of prostatitis.^47^ A recent human example is a prospective single-arm pre-post Li-ESWT study, in which symptom improvement was accompanied by measurable shifts in the gut microbiome.^61^ Another human MR study further supported a possible causal link between the gut microbiota and prostatitis risk, with the order of gastroanaerobes showing a positive association with prostatitis.^82^^,^^83^ Genetic evidence can strengthen causal inference and complement observational studies and animal-model findings. Animal models have identified mechanisms involving barrier integrity, the Th17 axis, and metabolite-mediated immune regulation, while human studies have begun to detect corresponding signals in patients. Bidirectional validation between experimental work and clinical studies can translate mechanistic insights into testable targets and improve the interpretability of model-based findings. Overall, integrating cross-species evidence strengthens the biological rationale for the gut–prostate axis as an entry point for CP/CPPS intervention.

### Neuroimmune modulation of the gut–prostate axis

As discussed above, neurogenic inflammation, central sensitization, and psychosocial stress are not alternative explanations that exclude the gut–prostate axis; rather, they may shape how gut-derived immune or metabolic signals are perceived, amplified, and maintained as chronic pelvic pain. In this sense, neuroimmune signaling should be viewed as a modulatory layer within the Dual-Hit Permissive Model rather than as a separate unidirectional brain-gut-prostate causal chain.^33^

Several neuroanatomical pathways may connect pelvic inflammation, gut function, and pain amplification. Peripheral nociceptive input from the prostate, pelvic floor, bladder, or adjacent pelvic organs can be transmitted through pelvic, pudendal, hypogastric, and splanchnic afferents to dorsal root ganglia and spinal dorsal horn circuits. Repeated input may promote peripheral sensitization, dorsal horn amplification, glial activation, and altered descending pain modulation. Autonomic pathways may also contribute: Sympathetic and parasympathetic outputs can influence prostate smooth-muscle tone, pelvic organ function, gut motility, immune tone, and intestinal permeability. In parallel, stress-responsive HPA-axis and CRF-related signaling may link psychological stress to permeability changes, immune activation, and pain persistence.^79^^,^^84^^,^^85^

Human neuroimaging studies provide further support for central nervous system involvement in CP/CPPS. Farmer et al. reported brain functional and anatomical changes in men with CP/CPPS.^15^ In the MAPP Network, resting-state functional connectivity measures predicted longitudinal pain symptom change in urologic CPPS, supporting the clinical relevance of central pain-network features.^86^ These findings do not prove that microbiome alterations drive central sensitization, but they show that prostate and pelvic pain symptoms are embedded within broader brain-network and pain-modulatory systems.

Neuromodulation studies also support the clinical relevance of neural pathways in male CP/CPPS. For example, posterior tibial nerve stimulation has been reported to relieve pain in category IIIB chronic nonbacterial prostatitis/CPPS.^87^ Reviews of neuromodulation in male chronic pelvic pain further suggest that sacral, tibial, or related nerve-targeted approaches may benefit selected patients, although evidence remains limited and heterogeneous.^88^ These interventions should not be interpreted as microbiome-targeted therapies. Instead, they indicate that pain-network and autonomic pathways can be clinically relevant modulators of CP/CPPS symptoms.

Within the Dual-Hit framework, neuroimmune signaling can influence both hits. Stress and autonomic dysregulation may worsen intestinal permeability, alter microbial ecology, or amplify systemic inflammatory tone, thereby modulating the first hit. At the same time, pelvic afferent sensitization, dorsal horn amplification, and prostate-local neurogenic inflammation may lower the threshold for pain persistence and inflammatory amplification, thereby modulating the second hit. Thus, the neuroimmune component provides a bridge between gut-derived immunometabolic priming, prostate-local permissiveness, and chronic pelvic pain, while remaining a testable modulatory loop rather than an established linear causal pathway.

## Clinical implications and therapeutic potential

### Translational framing

Recognition of the gut–prostate axis in CP/CPPS should be viewed as a source of translational hypotheses rather than as a mature therapeutic framework. Current human evidence remains limited, heterogeneous, and sometimes inconsistent, whereas many mechanistic links are supported primarily by animal prostatitis models or contextual prostate-disease studies. Therefore, gut-directed interventions should not be presented as established disease-modifying therapies for CP/CPPS. Instead, they should be evaluated as mechanism-based adjunctive strategies in biomarker-defined subgroups.^39^^,^^89^

A rigorous translational framework should require three levels of evidence. First, stratified human evidence is needed to identify patient subgroups in whom gut-related mechanisms are biologically plausible. Second, causal validation should come from longitudinal studies, microbiota-depletion or recolonization models, FMT-based transfer experiments, MR, and controlled intervention studies. Third, target engagement should be demonstrated by changes in permeability markers, microbial metabolites, immune phenotypes, or prostate-local inflammatory readouts that align with symptom trajectories. By this stricter standard, most microbiome-focused strategies should currently be considered investigational or complementary approaches requiring validation in biomarker-stratified trials.

### Biomarker stratification

A key translational distinction is that “measurable” does not mean “clinically validated.” Several candidate biomarkers can already be measured in research or specialized clinical settings, including stool microbial composition or metagenomic function, serum LPS-related or permeability-associated markers, inflammatory cytokines, T cell phenotypes, and stool or circulating microbial metabolites. Human CP/CPPS microbiome studies support the potential value of fecal microbial signatures as non-invasive biomarkers, but existing cohorts remain modest and heterogeneous.^59^^,^^60^^,^^62^ Similarly, serum LPS-related markers, bile acid profiles, SCFA-related readouts, indole metabolites, and Th17/Treg-associated immune phenotypes can be quantified, but none has yet been validated as a routine clinical biomarker for selecting gut-directed therapy in CP/CPPS.

Several biomarkers central to the Dual-Hit Permissive Model remain experimental. These include shared TCR clonotypes across gut, blood, and prostate-local compartments; mucosal-homing receptor signatures in prostate-infiltrating immune cells; spatially resolved prostate endothelial activation; MAdCAM-1 or mucosa-like addressins; and paired gut–blood–prostate single-cell or metabolomic profiles. These readouts are important for causal validation and source attribution, but they are not ready for routine clinical implementation. Therefore, biomarker-driven stratification should be presented as a research priority and trial-design principle rather than as an immediately translatable clinical strategy.

### Diagnostic implications

For clinicians, the immediate implication of the gut–prostate axis is not that microbiome testing should be used as a stand-alone diagnostic tool for CP/CPPS. Rather, gut-related features may eventually help refine patient phenotyping within existing multidomain frameworks.^13^^,^^14^ Patients with CP/CPPS who have gastrointestinal comorbidity, metabolic syndrome, repeated antibiotic exposure, diet-sensitive symptom fluctuation, elevated systemic inflammatory tone, or candidate permeability/metabolite abnormalities may represent a subgroup in whom gut-immune mechanisms deserve closer research attention.

At present, fecal microbial signatures, serum LPS-related markers, bile acid profiles, SCFA or indole readouts, and Th17/Treg-related immune phenotypes should be considered investigational adjuncts rather than validated diagnostic markers.^59^^,^^60^^,^^62^ Their potential value lies in future stratification: Identifying patients more likely to benefit from microbiome- or immunometabolism-informed trials, distinguishing gut-associated inflammatory phenotypes from predominantly neurogenic or psychosocial pain phenotypes, and selecting mechanistic endpoints for target-engagement studies.

### Microbiome-directed adjunctive strategies

Probiotics, prebiotics, synbiotics, and related microbiome-directed interventions have a mechanistic rationale because intestinal dysbiosis, barrier dysfunction, and Th17-type inflammation may be linked in a subset of patients with CP/CPPS. However, current human studies remain limited, and therapeutic responses are likely to depend on baseline microbiota composition, diet, gastrointestinal comorbidity, prior medication exposure, strain selection, dose, and formulation. These interventions should therefore be tested as hypothesis-based adjunctive strategies rather than recommended as general stand-alone treatments. Future trials should define responder hypotheses in advance, enroll or stratify patients with relevant baseline abnormalities, and document target engagement through metagenomic, metabolomic, permeability, or immune readouts before attributing symptom improvement to microbiome correction.

Diet is a flexible modulator of gut microbial and immunometabolic outputs, but it should also be treated as a testable mechanism-based approach rather than as a universal recommendation. High-salt models provide a rationale for testing diet-sensitive, microbiome-dependent immune pathways in biologically selected patients, but salt reduction, fiber enrichment, fat-quality modification, or polyphenol-based approaches should be evaluated prospectively with predefined target-engagement endpoints.^32^ Diet-related studies should also control for antibiotic exposure, metabolic status, gastrointestinal symptoms, and baseline dietary patterns, because these variables can strongly influence microbiome and immune readouts.

Antibiotics require a particularly cautious interpretation. Short antibiotic courses can improve symptoms in some patients with CP/CPPS, even when no active infection is detected, but this does not necessarily imply durable microbiome correction. Antibiotics may transiently reduce microbial ligand burden, alter bile acid or tryptophan metabolism, and modulate Th17-type inflammatory programs, yet they can also disrupt colonization resistance, impair microbial stability, and increase antimicrobial resistance risk.^90^ Therefore, antibiotic responses should be interpreted through responder phenotypes and paired biological readouts rather than used as evidence that broad microbiome resetting is clinically appropriate.

FMT is conceptually attractive for restoring dysbiosis, but CP/CPPS evidence remains insufficient for clinical translation. Case-level observations and animal-transfer experiments support causal plausibility, but controlled human trials with rigorous phenotyping, safety oversight, and mechanistic endpoints are still needed before FMT can be considered beyond research settings.^91^ In prostatitis models, transfer of dysbiotic microbiota has been associated with Th17 infiltration, macrophage polarization, and prostate inflammation, whereas transfer from treated donors can attenuate inflammatory readouts.^35^^,^^39^ These findings strengthen the rationale for microbiome restoration as an experimental strategy, but they do not establish FMT as a validated therapy for human CP/CPPS.

### Mechanism-specific experimental targets

Metabolite-focused strategies provide one route toward mechanism-specific intervention. SCFAs, tryptophan-derived indoles, bile acids, and related microbial metabolites can influence barrier function, Th17/Treg balance, inflammasome activity, and myeloid polarization. In principle, interventions could increase endogenous SCFA production through dietary fiber or prebiotics, promote microbial communities that generate beneficial metabolites, or modulate bile acid receptor pathways such as FXR and TGR5. However, metabolite-based interventions should be developed with standards similar to those used for pharmacological strategies: The relevant metabolic defect should be defined, the dose and route should be justified, circulating or stool metabolites should be quantified, and symptom improvement should be linked to restoration of the targeted pathway.

Immune-trafficking approaches are even more experimental. The gut–prostate axis framework suggests that systemic mucosal immune priming may converge with prostate-local recruitment signals, providing a rationale for targeting chemokine axes, S1P-dependent lymphocyte egress, or downstream IL-17/IL-1β inflammatory amplification.^6^^,^^35^ However, the dominant trafficking axis in human CP/CPPS remains undefined, and the upstream origin of prostate-infiltrating immune cells has not been established. Because chemokine and adhesion pathways are redundant and important for host defense, immune-trafficking interventions should remain experimental strategies requiring biomarker-defined patient selection, target-engagement validation, and careful safety assessment.

Contextual evidence from BPH and PCa supports the broader principle that microbiome-derived functions may influence prostate-relevant inflammation, metabolism, or endocrine signaling. For example, microbial metabolic pathways can affect androgen-related pools or inflammatory programs in PCa and BPH contexts.^69^^,^^92^ These observations do not provide direct evidence for CP/CPPS, but they support a function-first approach in which metagenomic pathway profiling and targeted metabolomics may be more informative than taxonomic associations alone.

### Neuroimmune and multidisciplinary strategies

As summarized in Figure 4, pain sensitization, stress-responsive signaling, and autonomic outputs may alter gut permeability, microbial ecology, immune tone, and pelvic pain amplification, potentially shaping symptoms in a subset of patients with CP/CPPS. Neuroimmune and stress-related pathways should therefore be integrated into the translational framework without being misclassified as microbiome-targeted therapies. Neuromodulation, behavioral strategies, multidisciplinary pain management, and stress-oriented interventions should be interpreted as adjunctive approaches targeting pain-network or autonomic components rather than as upstream microbiome-correction strategies.

Future neuroimmune studies should pair clinical endpoints with biological readouts. Relevant outcomes may include NIH-CPSI domains, pain durability, urinary symptoms, quality-of-life measures, stress metrics, autonomic markers, permeability readouts, inflammatory markers, and microbial or metabolite profiles. This design would allow investigators to distinguish symptomatic neuromodulatory benefit from true modification of gut-derived immunometabolic priming.

### Trial-design priorities

Overall, gut-directed therapeutic strategies in CP/CPPS should remain investigational or adjunctive until prospective studies demonstrate biomarker-defined responder subgroups, target engagement, and durable clinical benefit. This requirement applies equally to probiotics, diet, antibiotics, FMT, metabolite-focused approaches, immune-trafficking strategies, and neuroimmune interventions. The next stage of translation should therefore move from broad microbiome modulation toward mechanism-anchored trials that define the relevant subgroup, specify the intended biological target, confirm target engagement, and relate biological changes to clinically meaningful symptom improvement.

## Conclusions

The gut–prostate axis provides a useful framework for organizing emerging evidence in CP/CPPS, but its mechanistic and clinical significance remains to be validated. Current data support the plausibility of gut-derived immunometabolic priming and prostate-local inflammatory amplification, yet direct evidence for gut-origin prostate-infiltrating immune cells is still lacking. In particular, the absence of lineage-tracing, shared-TCR, and paired gut–blood–prostate immune-profiling data means that immune trafficking from the gut to the prostate should be regarded as a testable hypothesis rather than an established mechanism.

The Dual-Hit Permissive Model therefore should not be interpreted as a definitive explanation for all CP/CPPS cases. Instead, it offers a falsifiable roadmap for future studies by separating systemic immune priming from prostate-local permissive conditions. Future research should prioritize longitudinal human cohorts, MR-informed hypotheses,^58^^,^^93^ paired microbiome-metabolome-immune profiling, spatial analysis of prostate-local inflammatory signals, and biomarker-stratified intervention trials with clear target-engagement endpoints. Only through such causal and stratified validation can the gut–prostate axis be responsibly translated from a mechanistic hypothesis into a clinically useful framework.

## Acknowledgments

None.

Funding: This work was supported by grants from the 10.13039/501100001809National Natural Science Foundation of China (grant no. 82173121).

## Author contributions

S.P., Y.L., and K.S. conceptualized the review and designed its structure. S.P., Y.L., and Y.B. drafted the initial manuscript and prepared Figures 1, 2, 3, and 4 and Table 1. B.Y., Y.L., and S.W. performed literature searches, critically evaluated the referenced studies, and contributed key sections on mechanical signaling pathways, genitourinary tumor-specific landscapes, and therapeutic strategies. J.W. supervised the project, provided critical mechanistic and clinical insights, and revised the manuscript for intellectual content. All authors (S.P., Y.L., K.S., B.Y., S.W., Y.B., and J.W.) contributed to discussion of content, reviewed and edited the manuscript, and approved the final version submitted. J.W. and Y.B. are responsible for the overall content as guarantors.

## Declaration of interests

The authors declare no conflicts of interest.

## Declaration of generative AI and AI-assisted technologies in the writing process

The authors confirm that no generative AI or AI-assisted tools were used to create, modify, enhance, or reproduce any figures, graphical elements, artwork, or the graphical abstract in this Review. Figures 1, 2, 3, and 4 and the graphical abstract are original author-created schematic illustrations and were not directly reproduced from copyrighted publications.
